# Ultrapure Green High Photoluminescence Quantum Yield from FAPbBr_3_ Nanocrystals Embedded in Transparent Porous Films

**DOI:** 10.1021/acs.chemmater.3c00934

**Published:** 2023-07-07

**Authors:** Carlos Romero-Pérez, Natalia Fernández Delgado, Miriam Herrera-Collado, Mauricio E. Calvo, Hernán Míguez

**Affiliations:** † Instituto de Ciencias de Materiales de Sevilla (Consejo Superior de Investigaciones Científicas-Universidad de Sevilla), C/Américo Vespucio, 49, Sevilla 41092, Spain; ‡ Department of Material Science, Metallurgical Engineering and Inorganic Chemistry IMEYMAT, Facultad de Ciencias (Universidad de Cádiz), Campus Río San Pedro, s/n, Puerto Real, Cádiz 11510, Spain

## Abstract

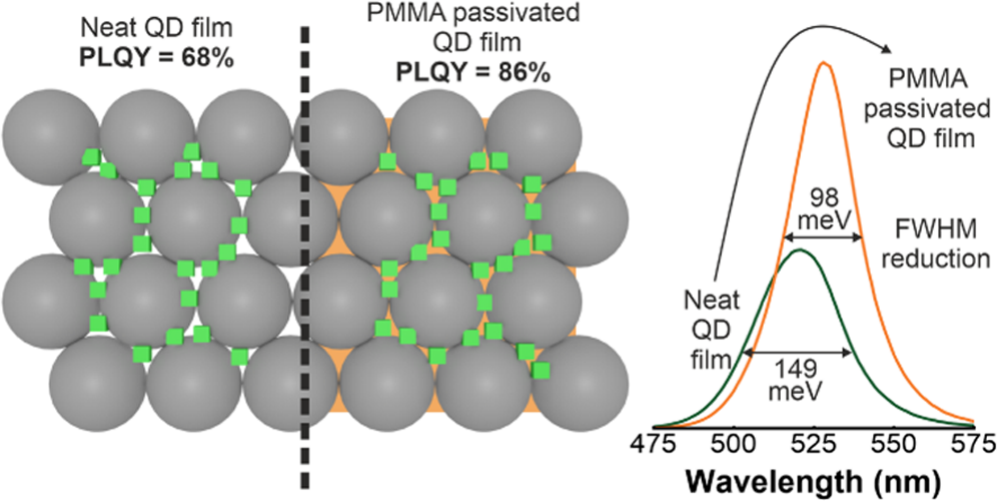

Achieving highly transparent and emissive films based on perovskite quantum dots (PQDs) is a challenging task since their photoluminescence quantum yield (PLQY) typically drops abruptly when they are used as building blocks to make a solid. In this work, we obtain highly transparent films containing FAPbBr_3_ quantum dots that display a narrow green emission (λ = 530 nm, full width at half-maximum (FWHM) = 23 nm) with a PLQY as high as 86%. The method employed makes use of porous matrices that act as arrays of nanoreactors to synthesize the targeted quantum dots within their void space, providing both a means to keep them dispersed and a protective environment. Further infiltration with poly(methyl methacrylate) (PMMA) increases the mechanical and chemical stability of the ensemble and serves to passivate surface defects, boosting the emission of the embedded PQD and significantly reducing the width of the emission peak, which fulfills the requirements established by the Commission Internationale de l’Éclairage (CIE) to be considered an ultrapure green emitter. The versatility of this approach is demonstrated by fabricating a color-converting layer that can be easily transferred onto a light-emitting device surface to modify the spectral properties of the outgoing radiation.

## Introduction

ABX_3_ perovskite nanocrystals^1,2^ have become central materials for the development of quantum dot (QD)-based optoelectronics. Record efficiency values have been attained in the field of QD-based photovoltaics,^3,4^ as well as very high external quantum efficiencies in the field of quantum light-emitting diodes (QLEDs),^5−7^ employing PQD as either light-absorbing or -emitting materials, respectively. PQDs display size confinement effects and low defect density, which allows achieving tunable and intense emission in wide spectral ranges,^8^ and present improved stability with respect to their bulk counterparts.^9^ The most extended method to prepare PQDs is colloidal synthesis, in which soluble precursors diffuse and react under mild conditions to obtain stabilized dispersions of PQDs with controlled and tunable size, composition, and shape.^10,11^ Within that approach, capping ligands play a central role to achieve defect passivation, by complexation of the surface, and chemical stability.^12,13^ Among the members of the ABX_3_ perovskite family, FAPbBr_3_ QDs are emblematic since they present outstanding stability^14,15^ and excellent emission properties,^16^ which lead to applications as either color-converting layers in displays^17−19^ or as electroluminescent materials in light-emitting devices (LEDs).^20,21^ However, in order to harness the potential of FAPbBr_3_ nanocrystals in optoelectronics, exhaustive and lengthy purification protocols (centrifugation, solvent replacement, ligand interchange, etc.) are required,^22^ which hinder their reproducibility. Furthermore, the processing of PQDs as thin layers must prevent aggregation and guarantee a homogeneous coverage of the substrate. These additional steps increase the duration and the cost of the total process. From the postprocessing perspective, even mild thermal treatments of FAPbBr_3_ QD films are detrimental to the optical properties.^23^

Alternatively, the preparation of perovskite nanocrystals can be also successfully attempted by employing porous materials as host scaffolds. As pointed out by Sanchez et al. in one of their earlier classical reviews,^24^ micro- and mesopores provide size and shape selectivity, as well as enhanced host–guest interactions, while the presence of macroporous channels permit improved access to the active sites at the immediate smaller scale, avoiding pore blocking by reagents or products. So as to apply this concept to the synthesis of perovskite nanocrystals, porous materials must present, preferably, a multiscale porosity, in order to optimize the accessibility and diffusion of perovskite precursors, and, at the same time, confine and stabilize the resulting nanocrystals in the smaller pores. The first attempts within this approach made use of mesostructured materials^25^ (i.e., ordered porous lattices with narrow pore size distribution and a well-defined pore shape and interconnectivity), yielding solid dispersions of more stable and efficient perovskite nanocrystals with a wide diversity of compositions, whose size distribution was determined by the voids of the pore network.^26−28^ Since then, several different synthetic routes have been proposed,^29−31^ with different mechanisms to control the nanocrystal size distribution occurring in each one of them. From a broader perspective, these approaches can be considered a particular example of synthesis based on nanoreactors, as it has been recently highlighted by Mirkin et al.^32^ In this context, the porous scaffold acts as a network of nanocavities in which perovskite precursors enter, react, and crystallize at the sub-10 nm scale. One key feature of the scaffold-supported nanocrystals is that their surface is not covered by ligands, as it happens in the case of colloidal particles. This opens the opportunity to realize clean studies of some fundamental interactions and properties, such as the electron–phonon interplay in perovskite nanocrystals,^33^ the determination of their optical constants,^34^ or their interaction with the environment,^35^ all this being possible due to the absence of surface-anchored organic ligands, always present in their colloidal counterparts. From a technological viewpoint, we could verify the successful operation of solar cells integrating insulating scaffold-supported nanocrystals, mediated by efficient dot-to-dot charge transport,^36^ as well as room temperature stabilization inside a porous structure of the highly unstable black phase of the CsPbI_3_ lattice,^37,38^ which in turn allowed developing optoelectronic devices based on it.^39^ Very recently, the potential offered by the interplay between the adsorption–desorption properties of the porous host and the emission of stabilized CsPbBr_3_ quantum dot guests was taken advantage of developing novel approaches to sensing.^40^ Overall, one of the main advantages the scaffold-supported approach endows is the opportunity to obtain a highly emissive transparent film without lengthy processing. However, the fact that no ligands were employed in the synthesis, although it allows performing fundamental studies otherwise impossible to make and favors charge transport, also implies that surface defects are not passivated, thus limiting the maximum PLQY that could be attained (on the order of 50%) and its monochromacity (typical peak widths in the range 120–150 meV).

In this work, we demonstrate a scaffold-assisted route to synthesize film-embedded FAPbBr_3_ QDs with size-tunable emission wavelength with a considerably high initial PLQY of 68%, which we improve up to 86% by post-treating the ligand-free nanocrystals with poly(methyl methacrylate) (PMMA). This enhancement is accompanied by a significant reduction of the PL spectral width (from 0.149 to 0.098 eV for a green emission at *h*ν = 2.34 eV or λ = 529 nm), thus increasing its monochromaticity. We also prove that the FAPbBr_3_ QD-embedding films can be used as highly efficient color converters by attaching them to commercial blue-light-emitting devices. Overall, these results prove that the scaffold-assisted synthesis of ABX_3_ perovskite QDs gives rise to light-emitting films with efficiencies comparable to the highest ones achieved from solid colloidal quantum dot solids and confirm their potential to be employed as a versatile color tuning tool in lighting technology.

## Results and Discussion

**Figure 1:**
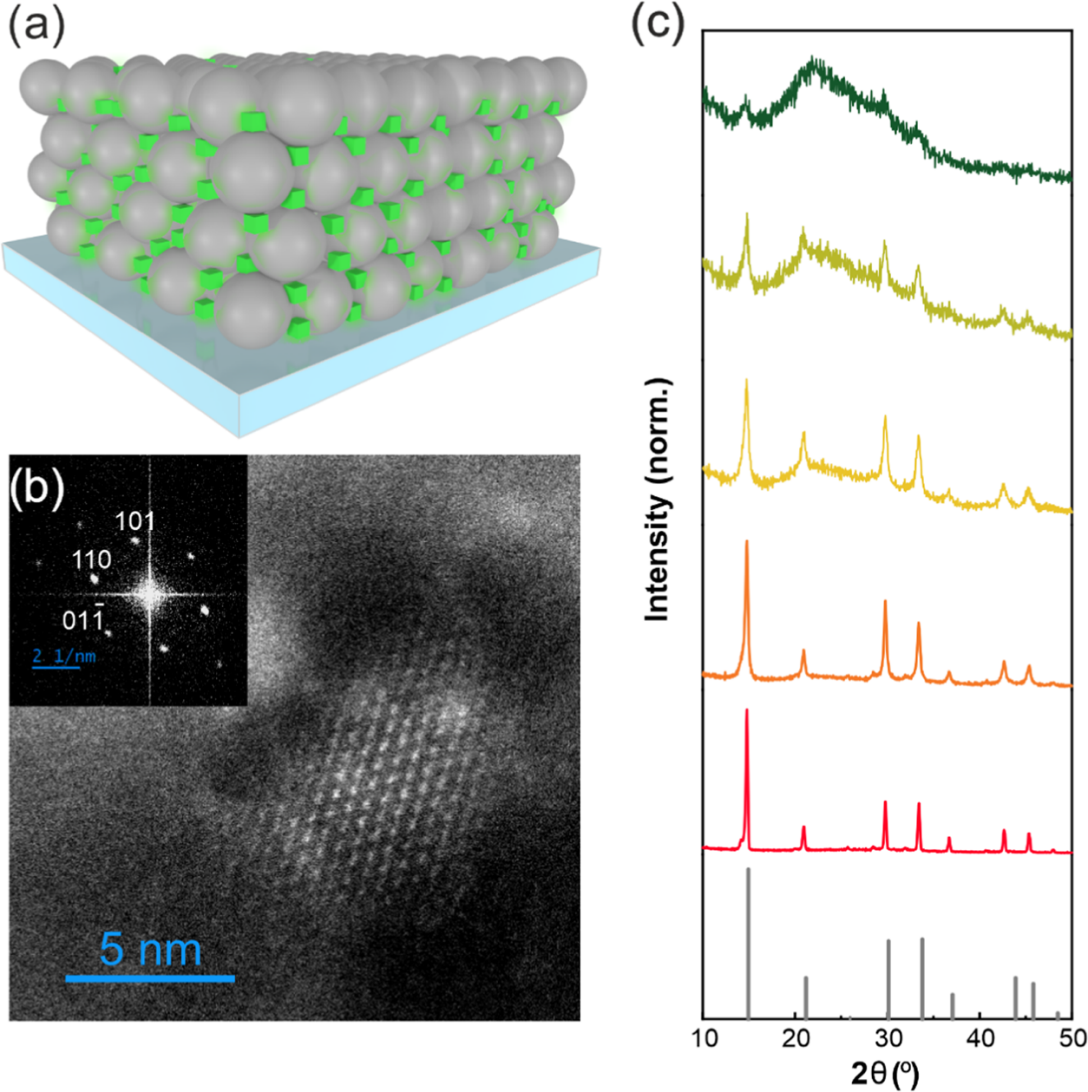
(a) FAPbBr_3_ QD-SiO_2_ film model. (b) High-resolution high-angle annular dark-field scanning transmission electron microscopy (HAADF-STEM) micrograph of a FAPbBr_3_ QD-SiO_2_ film lamella with a particle size around 8 nm. Inset shows the fast Fourier transform (FFT) image from the particle. (c) Normalized X-ray diffraction (XRD) diffractograms of FAPbBr_3_ QD@SiO_2_ films with different crystal sizes determined utilizing the Brus equation (8.7 olive, 10.2 light green, 11.0 yellow, 13.3 nm size orange) and bulk film (red).

Insulating SiO_2_ porous thin films with narrow pore size distribution was employed as scaffolds to synthesize FAPbBr_3_ nanocrystals that display well-defined quantum size effects. Porous films are made by dip-coating a colloidal suspension containing 30 nm size SiO_2_ spherical particles dispersed in methanol and water. After a thermal treatment at 450 °C, which eliminates solvent and organics from the porous matrix, FAPbBr_3_ precursors dissolved in dimethyl sulfoxide (DMSO) are infiltrated by spin-coating, i.e., dropping the solution onto the porous film and subsequently spinning the substrate to prevent the formation of a bulk perovskite overlayer onto the film. A posterior thermal treatment removes DMSO and allows the crystallization of FAPbBr_3_ within the pore network of the SiO_2_ film. Full details can be found in the Experimental Section. The scheme in Figure 1a represents the FAPbBr_3_ nanocrystal inclusions (green cubes) resulting from the infiltration and crystallization of the precursors in the packed SiO_2_ nanoparticle matrix. Direct visualization of the dispersion of FAPbBr_3_ QDs was achieved by observing the cross sections of lamellae under a high-resolution transmission electron microscope (HRTEM), as shown in Figure S1. High-resolution images of the cross section of a film allow direct observation of the silica-scaffold-supported perovskite nanocrystals, revealing their high crystallinity, as in the example shown in Figure 1b. Control over the average nanocrystal size was achieved by gradual variation of the perovskite precursor concentrations, following a procedure that has been applied before for other compositions.^34^ A collection of X-ray diffractograms of all QD sizes prepared for this work are displayed in Figure 1c. In all cases, 2θ positions of the peaks show good agreement with those of the cubic phase FAPbBr_3_ standard (gray line).^41^ Deposition of a homogeneous bulk film using the same precursors yields this same phase (red line). As expected, increasing the concentration of the precursors, which implies increasing the average nanocrystal size, correlated with the narrowing of the X-ray diffraction peaks of the different SiO_2_-containing FAPbBr_3_ nanocrystals. From Brus formula^42^
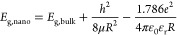
1we estimate the size of the FAPbBr_3_ crystals as 8.7, 10.2, 11.0, and 13.3 nm for 5, 10, 20, and 40% (w/w) concentration, respectively. Bearing in mind that the reported Bohr radius for this FAPbBr_3_ is around 8 nm,^43^ we can consider all of these different nanocrystals to be within a strong quantum confinement regime.^44^

**Figure 2:**
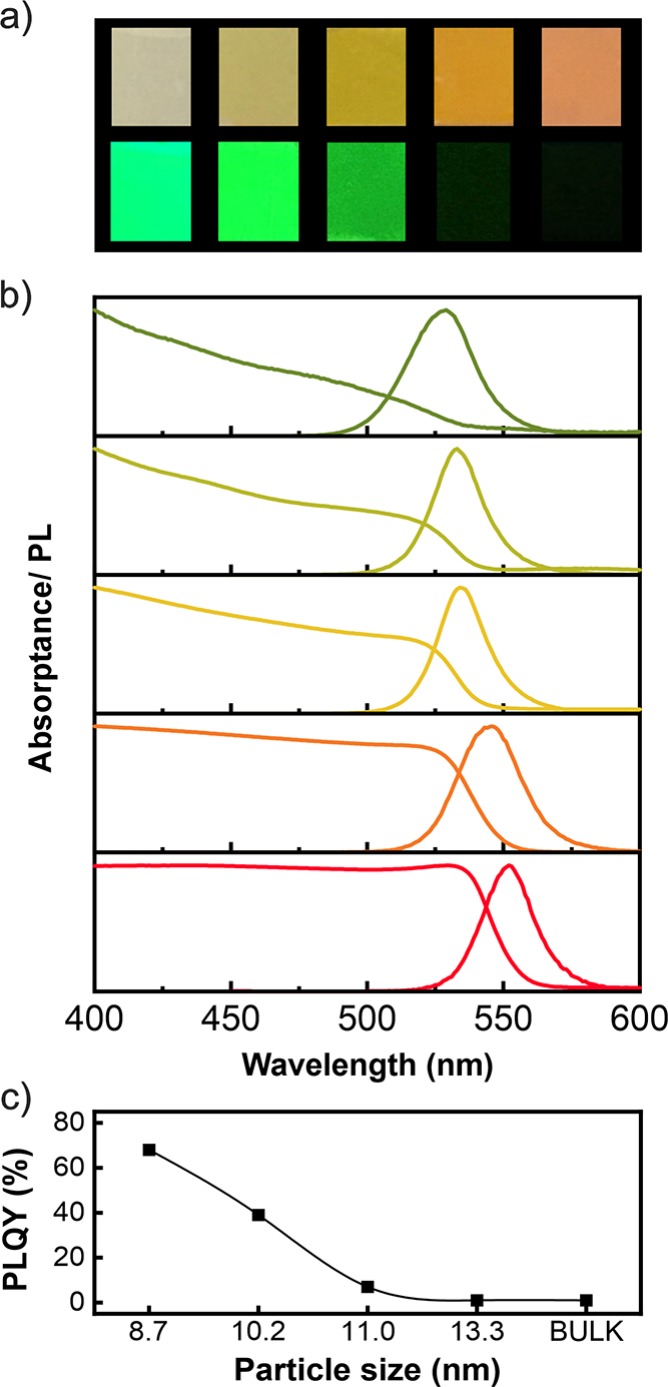
(a) Pictures of FAPbBr_3_ QD@SiO_2_ films with different crystal sizes (increasing from left to right) under diffuse white-light illumination (upper row) and inside a UV chamber under 365 nm wavelength excitation (lower row). (b) Absorptance and normalized PL spectra of FAPbBr_3_ for each one of the FAPbBr_3_-QD@SiO_2_ films under study. From top to bottom, the average nanocrystal size is 8.7 nm (olive curves), 10.2 nm (green), 11.0 nm (yellow), 13.3 nm (orange), and bulk (red curves). (c) PLQY versus average QD size.

Images of a collection of samples containing FAPbBr_3_ nanocrystals with different sizes illuminated under room light (upper row) and under UV light of λ = 365 nm in a dark chamber (lower row) are shown in Figure 2a. The homogeneous color demonstrates that the high optical quality of the scaffold is preserved after the infiltration and crystallization of FAPbBr_3_ QDs. Bright emissions are achieved for the lower perovskite filling fractions of the pores between3 and 8% of the pore volume. For higher values, PL gradually decreases until it is almost completely quenched in the bulk film. In Figure 2b, the normalized PL curves along with the absorptance corresponding to the samples shown in Figure 2a are plotted (raw PL spectra may be consulted in Figure S2). The PLQY measured from the FAPbBr_3_ QD@SiO_2_ films is shown in Figure 2c. It can be seen that the PLQY reaches up to 68% for the smaller nanocrystal sizes obtained. The high spatial dispersion of the nanocrystals within the porous structure, with a filling fraction of around 3% vol. of the pore according to measurements performed by inductively coupled plasma atomic emission spectroscopy (ICP-AES) (Figure S3), contributes to this high value, since, at this degree of infiltration, intergrain contacts between PQDs are less frequent, which prevents PL deactivation by carrier scavenging. Interestingly, along with the concentration, the PLQY can be optimized by varying the conditions of the spin-coating protocol and the crystallization temperature. The dependence of the photoemission efficiency on these parameters is included as the Supporting Information (Figures S4 and S5).

**Figure 3:**
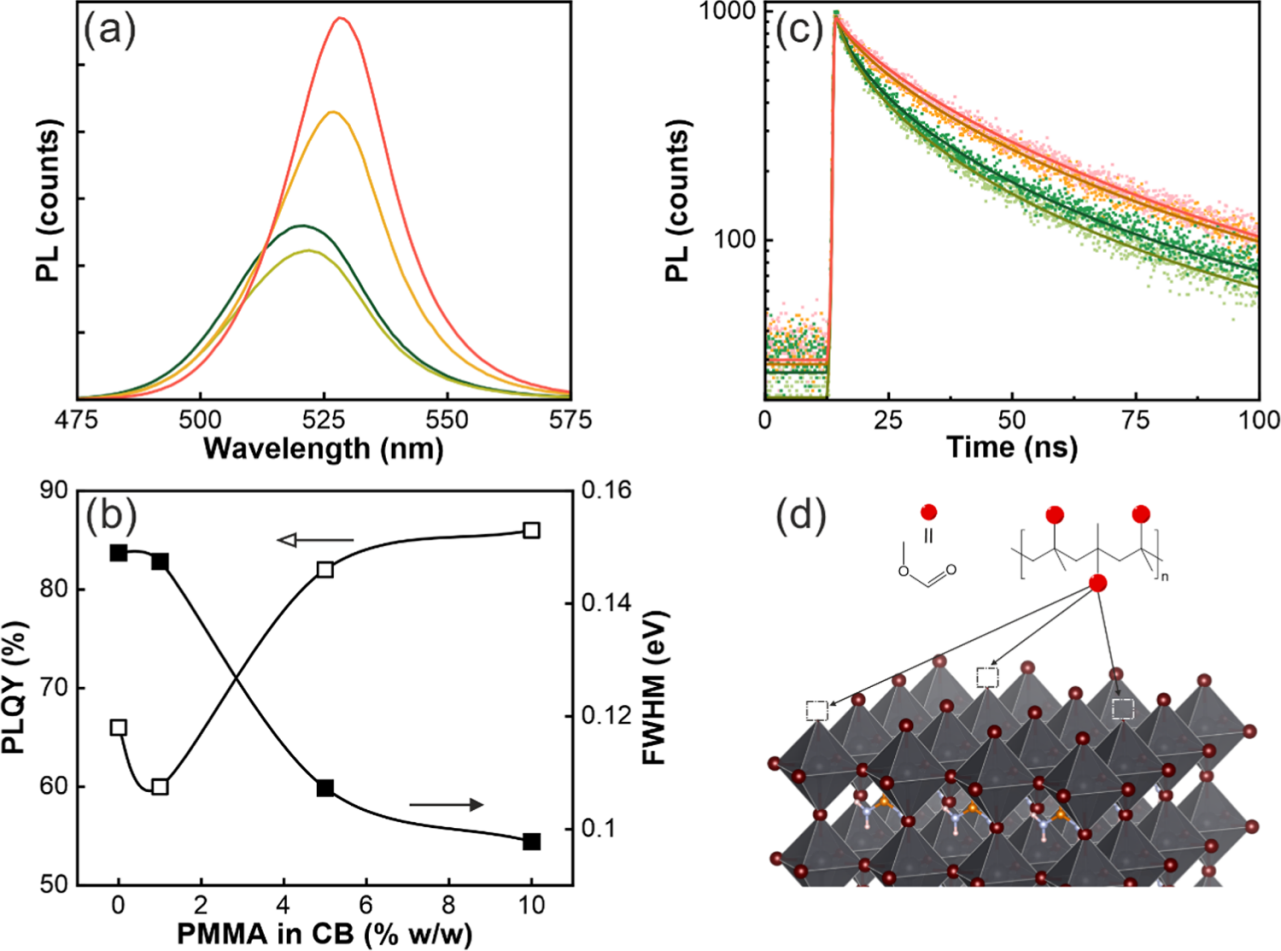
(a) PL, (b) PLQY (open squares) and FWHM (black squares), and (c) time-resolved PL of 8.7 nm FAPbBr_3_ QD@SiO_2_ infiltrated with different PMMA concentrations (% w/w) in CB. In panels (a) and (c), results for each PMMA concentration are identified with the following color codes: 0% dark green, 1% light green, 5% yellow, and 10% pale red. In panel (b), the line connecting the dots is just a guide to the eye. PL decays in panel (c) are fitted using a stretched-exponential decay model (solid lines). (d) Model of the passivation mechanism by which PMMA neutralizes uncoordinated Pb^2+^ sites represented by the empty square located at the top corner of the octahedra.

In order to further increase the light-emitting efficiency of the performance of the FAPbBr_3_ QD@SiO_2_ films, we explored the possibility of processing our materials using a passivating agent. We were motivated by the fact that not all the void space within the porous scaffold is filled by the perovskite, as indicated by the ICP-AES measurements, which gives us the chance to perform a second infiltration. Also, in a previous study, we had observed an increase of the PLQY of methylammonium lead bromide, MAPbBr_3_, nanocrystals embedded in a porous scaffold after the subsequent infiltration with an elastomer, poly(dimethylsiloxane),^36^ which we mainly attributed to an effect of an increase of the local density of photon states as a result of the larger refractive index.^45^ Among many different compounds that have been employed to reduce the effect of surface carrier traps, poly(methyl methacrylate) (abbrev. PMMA) has been shown to provide both physical isolation and chemical passivation. Also, it is used as an interlayer in optoelectronic devices to efficiently protect the perovskite film surface against moisture, improving the conversion efficiency and the stability against humidity.^46−49^ Also, PMMA is easily dissolved in orthogonal solvents (such as chlorobenzene, CB), i.e., those that do not dissolve the ABX_3_ perovskite, hence not affecting the stability of the synthesized FAPbBr_3_ QD. With these precedents in mind, PMMA solutions in CB at different concentrations were infiltrated within the remaining void space of a FAPbBr_3_-QD@SiO_2_ film fabricated by spin-coating, and subsequently the film was dried at 90 °C to evaporate the remaining solvent. This process has barely any effect on the transparency of the film but a strong impact on the photoemission efficiency. In Figure 3a, a significant increase in PL intensity, along with a gradual red shift of the PL peak spectral position, is observed as we increase the concentration of PMMA. For the largest PMMA concentration, we achieved a remarkable PLQY of 86% (Figure 3b), which is among the largest values reported for FAPbBr_3_ QD films,^7,50^ being all previously reported obtained by depositing colloidal nanocrystals. The increase of PLQY is accompanied by a narrowing of the PL maximum, its full width at half-maximum (FWHM) being lowered from 149 meV in the initial FAPbBr_3_ QD@SiO_2_ film to 98 meV in that with the highest degree of PMMA infiltration.

Let us analyze the origin of these changes, starting with the significant PLQY enhancement observed for the polymer-embedded samples. The PL decay dynamics, disclosed in Figure 3c, reveals that, upon PMMA infiltration, the excited state lifetime is dramatically increased 230% with respect to the initial value (Table S1). This observation supports the hypothesis of PMMA acting as a passivating agent of the ligand-free FAPbBr_3_ QD embedded in the porous film, hence lowering the nonradiative decay rate (Γ_NR_) and increasing the lifetime of the excited state. In this case, this effect is prevailing over the also expected increase of the radiative decay rate (Γ_R_) caused by the larger dielectric constant surrounding the emitter.^41^ All of these observations point at a passivating effect of PMMA, which has been reported to coordinate through carboxylic groups to Pb^2+^ centers,^51^ which act as nonradiative carrier recombination centers at the surface of perovskite grains.^52,53^ A scheme of the proposed passivation mechanism is shown in Figure 3d. Regarding the red shift of the PL peak reported in Figure 3a, it can be attributed to the expansion of the orbitals toward the PMMA layer surrounding the nanocrystals, which gives rise to an effective reduction of the exciton confinement, as it has been proposed for organic-capped QDs.^54^ This hypothesis is further confirmed by analysis of the PL intensity temperature dependence, as shown below. Concerning the dramatic and intriguing spectral narrowing of the PL peak observed, as PL spectral broadening is typically determined by inhomogeneous energetic disorder, and since PMMA cannot affect the QD size dispersion present in the film, we can conjecture that peak width reduction may be attributed to a decrease, as a result of the presence of an insulating PMMA coating, of nonuniformly distributed short-range interactions with neighboring nanocrystals.

**Figure 4:**
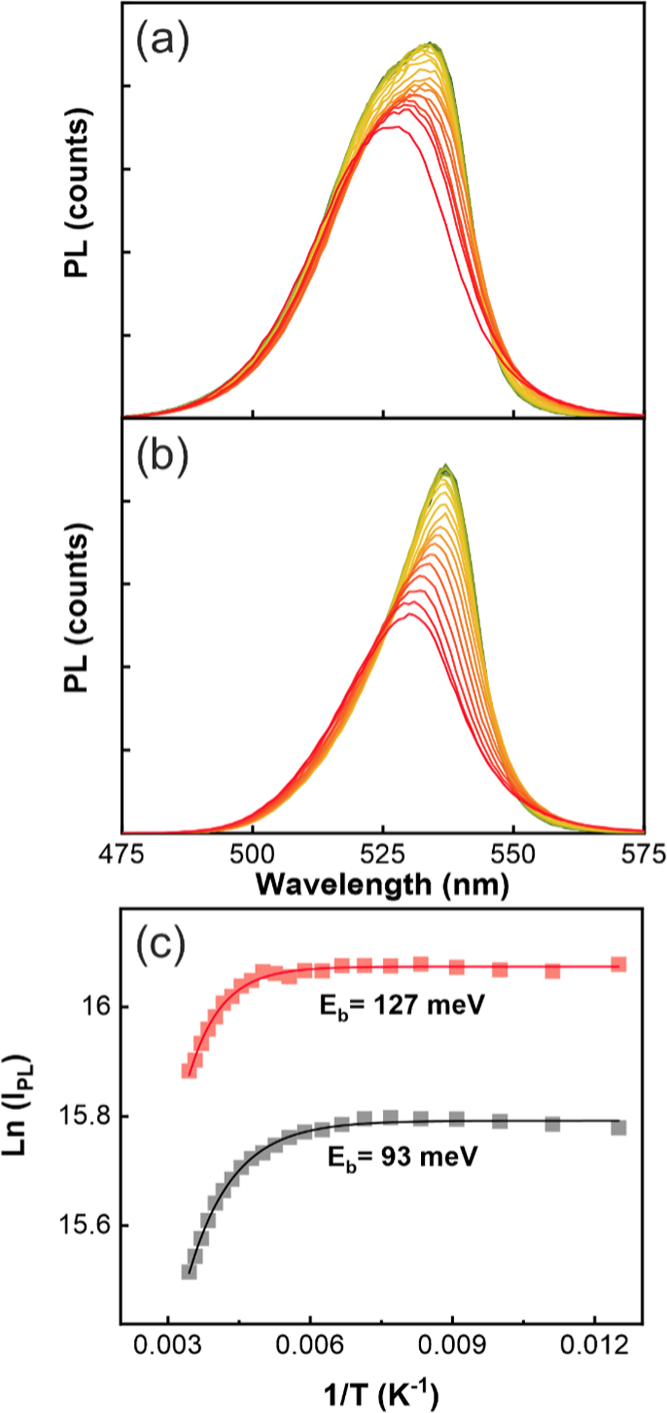
Temperature-dependent PL of 8.7 nm FAPbBr_3_ QDs embedded in a porous SiO_2_ film (a) before and (b) after PMMA infiltration (10% w/w), measured in the range comprised between 290 K (red line) and 80 K (green line). (c) Logarithm of the integrated PL intensity before (red squares) and after PMMA infiltration (gray squares) versus the inverse of the temperature. Fittings to Arrhenius equations are also plotted (red and gray solid lines, respectively) and the estimated value of *E*_b_ indicated in each case.

Further insight into the effect of PMMA infiltration within the FAPbBr_3_ QD@SiO_2_ films is gained by measuring the temperature dependence of PL. In Figure 4, we plot the PL spectra attained at temperatures comprised between 80 and 290 K for QD films before (Figure 4a) and after (Figure 4b) adding PMMA. The red shift of the PL peak as the temperature decreases, observed in both cases, is characteristic of ABX_3_ perovskites and a consequence of the antibonding character of the electronic orbitals involved in the formation of the valence and conduction bands, as it has been described before.^55^ We can extract information on the binding energy of the exciton by fitting the measured temperature variation of the integrated photoemission intensity, *I*(*T*), plotted in Figure 4c, using an Arrhenius equation^56^
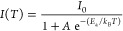
2where *I*_0_ is the integrated PL intensity at 0 K, *A* is the Arrhenius constant, *E*_a_ is the activation energy of the photoemission process, and *k*_B_ is the Boltzmann constant. The corresponding fittings to eq 2 are plotted as red and gray solid lines for neat and PMMA-passivated QDs, respectively. Assuming that the exciton binding energy, *E*_b_, approximately equals *E*_a_, we obtain *E*_b_ = 127 meV for the bare nanocrystals and *E*_b_ = 93 meV after PMMA infiltration, further revealing the strong effect of this passivating agent on the exciton properties. The *E*_b_ estimated for bare nanocrystals concurs with that previously reported for colloidal nanocrystals with a similar composition and size^57^ and largely exceeds the values determined for bulk FAPbBr_3_,^58^ as it has been observed for confined excitons in other semiconductors.^59^ These results are in good agreement with the observed PL red shift and the consequently proposed reduction of the exciton confinement due to the presence of a PMMA coating around the nanocrystals.^60^ As the crystal size of FAPbBr_3_ QDs in the film increases, the effect of PMMA addition on the *E*_b_ is vanished, as shown in Figure S6, probably as a result of higher density of bulk defects in larger crystals, which makes them less sensitive to the surface passivation effect of the polymer. It should be kept in mind that eq 2 neglects the existence of recombination pathways other than radiative ones, which may limit the validity of the approximation herein employed.^61−63^ In our case, the comparison is established between emitters for which radiative decay clearly dominates the de-excitation process, as demonstrated by the high PLQY of both the bare and the PMMA-infiltrated films. Interestingly, the analysis of the spectral position of the PL peak for the larger QD sizes (approximately between 10 and 11.0 nm) prepared reveals subtle changes occurring near 160–180 and 250–270 K (Figure S7). These changes can be attributed to crystalline phase transitions occurring at each temperature interval, likely to the orthorhombic (γ, *Pnma*) to tetragonal (β, *P*4/*mbm*) and tetragonal to cubic (α, *Pm*3̅*m*) transitions, as it has been reported for bulk FAPbBr_3_. These changes are seemingly absent in the temperature-dependent curves obtained for the smaller QDs, in good agreement with previous works.^64,65^

**Figure 5:**
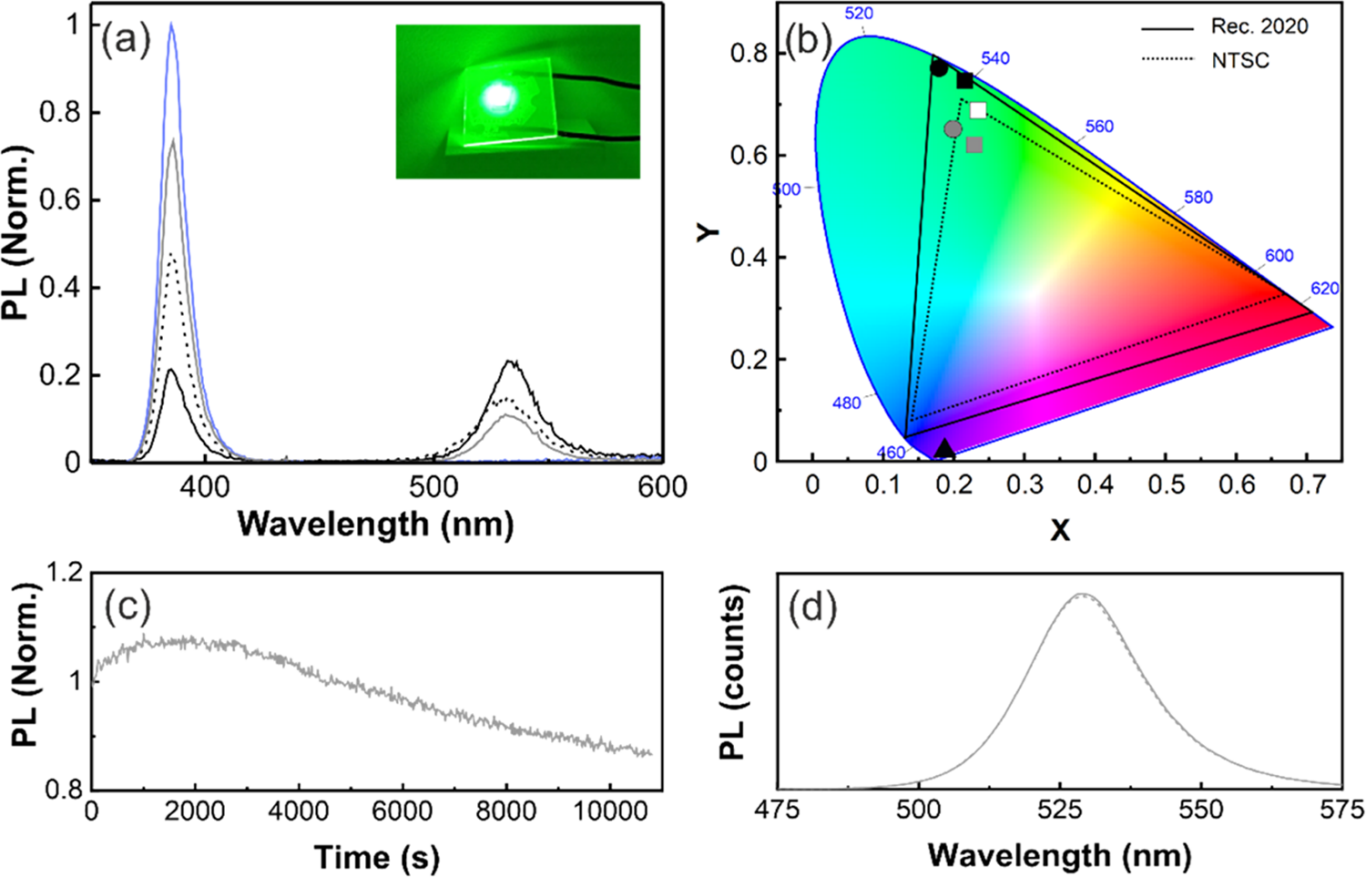
(a) Conversion of the λ = 385 nm emission of a GaN LED chip (violet line) to green by PMMA-infiltrated FAPbBr_3_ QD@SiO_2_ films with QD sizes of 8.7 nm (solid gray line) and 10.2 nm (black lines); in this latter case, the curves attained for 1 and 2 μm thick porous scaffolds are depicted (dotted and solid black lines, respectively). (b) Correspondingly, CIE coordinates from the PL spectra of bare GaN LED (black triangle); FAPbBr_3_ QD@SiO_2_ films with QD sizes of 8.7 nm (black circle) and 10.2 nm (black square) pure PL and after violet light conversion (gray circle and square). 2 μm thick scaffold is represented as a hollow square. (c) PL stability under continuous irradiation of a FAPbBr_3_ QD@SiO_2_ film infiltrated with PMMA. (d) Initial (solid line) and final PL spectra (dashed line) acquired before and after the stability measurement.

The quality, applicability, and versatility of the PMMA-passivated FAPbBr_3_ QD@SiO_2_ films as color-converting layers for lighting devices were tested by adapting them to the flat surface of a GaN LED chip with a violet emission peak centered at 385 nm. In order to do so, we used thicker scaffolds loaded with FAPbBr_3_ QDs, ensuring both a sufficient absorptance of the excitation (28% < *A* < 81% at λ = 385 nm) while preserving a considerable PLQY (40% < PLQY < 86%). In Figure 5a, the degree of color conversion achieved with different FAPbBr_3_ QD@SiO_2_ film thicknesses and QD sizes is illustrated. These results show that just a 2 μm thick (see Figure S8) color-converting PMMA-infiltrated FAPbBr_3_ QD@SiO_2_ film (black solid line) is enough to reach a 1:1 violet-to-green ratio, confirming that color conversion can be reasonably controlled with the available experimental parameters. Interestingly, the analysis of the corresponding coordinates in the CIE 1931 color space, shown for a range of samples in Figure 5b, demonstrates that the narrow emission peak obtained after PMMA addition, centered in λ = 529 nm and characterized by coordinates (*x* = 0.1733, *y* = 0.7644), practically meets the requirements to be considered an ultrapure green emission, established at (*x* = 0.170, *y* = 0.797), which is considered as the key for the development of next-generation screens.^6,53^ Also, PMMA-embedded scaffold-supported FAPbBr_3_ QDs show enhanced long-term photostability. To study this, PL measurements, using a λ = 430 nm excitation line, were carried out under continuous irradiation (≅2 mW/cm^2^) for 3 h. Results shown in Figure 5c,d disclose a transient behavior, typical of solution-processed organic lead halide perovskites,^30,66,67^ and characterized by a photoactivation period, which in this case takes place during the initial 30 min, followed by a slow deactivation. After 3 h, PL still preserves 90% of the initial intensity. Furthermore, by keeping the irradiated sample in the dark for 2 min, the original PL spectrum is fully recovered, showing that no irreversible damage (i.e., no degradation) was caused to the QD during the entire irradiation process. Stability versus relative humidity (RH) in the 40% < RH < 80% range was also studied. Moisture was varied in the sample chamber by combining dry and wet nitrogen flux, and the evolution of PL vs time was monitored at each RH value. Results are provided in Figure S9. For RH ≤ 55%, the PL stands almost unaltered for the whole duration of the experiment, and in all cases, the exact same PL spectrum is recovered after removing the sample from the chamber. Only for the highest RH tested (i.e. RH=80%), a 10% drop of the PL is observed after 3 h, which in this case is irreversible. This stability may be attributed to the protective environment provided by the porous SiO_2_ scaffold infiltrated with PMMA, which prevents diffusion of water or oxygen into the inner space of the film in which QDs are embedded.

## Conclusions

We have demonstrated a synthetic route to attain transparent films displaying highly efficient ultrapure green emission from ligand-free FAPbBr_3_ quantum dots embedded in nanoporous scaffolds. Quantum yields as high as 86% were achieved as a result of sequential infiltration of PMMA in the remaining void space of the matrix. This postsynthesis defect passivation effect allows reaching the quantum yield values hardly achievable by any other method without sacrificing the transparency of the film. The advantage and versatility of these materials when integrated as color-converting layers into LED technology were also shown, as well as the enhanced photostability provided by the combined protective effect of the porous matrix and the infiltrated polymer.

## Experimental Section

### Materials

Formamidinium bromide (FABr, GreatCell Solar Materials, 99,9%), lead(II) bromide (PbBr_2_, TCI, 99,99%), dimethyl sulfoxide (DMSO, Merck, anhydrous 99.8%), methanol (MeOH, VWR, 98%), poly(methyl methacrylate) (PMMA, Alfa Aesar, 99.9%), and chlorobenzene (CB, Merck, 99.9%) were purchased and used without additional purification steps.

### Preparation of SiO_2_ Nanoparticle Porous Scaffold

A commercial colloidal suspension of 30 nm SiO_2_ nanoparticles (34% w/v in H_2_O, LUDOX-TMA, Sigma-Aldrich) was diluted in methanol to 3% w/v. This diluted suspension was dip-coated on top of a low-fluorescence glass substrate employing a 120 mm/min withdrawal speed. Deposition was repeated 15 times in total to produce a porous scaffold of thickness around 1 μm, which was then annealed at 450 °C for 30 min both to remove any organic component remaining within the matrix and to improve its mechanical stability. A 2 μm thickness film was made depositing an initial film with 11 layers and annealing it at 450 °C for 30 min to repeat the deposition procedure of 11 layers, followed by a final treatment at 450 °C.

### Synthesis of FAPbBr_3_ Nanocrystals within Nanoporous Silica Scaffold

A perovskite solution precursor was prepared using FABr and PbBr_2_ powders in a 1:1 molar ratio in DMSO at different concentrations. Infiltration of this solution within the void space of the scaffold was performed via spin-coating (5000 rpm for 60 s), followed by heating at 100 °C for 1 h, to obtain FAPbBr_3_ nanocrystals within the pores of the matrix. Solutions in DMSO were prepared with concentrations comprised between 40 and 5% w/w.

### PMMA Infiltration

Solutions of different concentrations of a poly(methyl methacrylate) precursor (1, 5, and 10% w/w) were prepared by dissolving PMMA in chlorobenzene at 60 °C; 200 μL of this solution was dropped onto the nanocrystal-embedded porous scaffold and then spin-coated at 5000 rpm for 1 min. A final annealing step at 90 °C to cure PMMA was carried out. Every step related to the synthesis of FAPbBr_3_ and further PMMA infiltration was conducted inside a nitrogen-filled glovebox.

### Structural and Compositional Characterization

HAADF-STEM micrographs were acquired from lamellae obtained with a focused-ion beam (FIB) instrument (Scios 2 DualBeam, Thermo Fisher Scientific) using an FEI Titan Cubed Themis (scanning) transmission electron microscope operated at 200 kV. X-ray diffractograms were collected in a Philips X’pert PRO X-ray diffractometer utilizing Cu Kα radiation (λ = 1.54518 Å) in an acquisition range (2θ) between 10–50° with a 0.05° step. Perovskite pore filling fraction (PFF) determination was performed by inductively coupled plasma atomic emission spectroscopy (ICP-AES) using 10 mL of water solutions of soaked FAPbBr_3_ QD-SiO_2_ PMMA films for 1 h (complete extraction of the perovskite in the film) in an iCAP 7200 ICP-AES Duo (Thermo Fisher Scientific) instrument for measurements.

### Optical Characterization

Photoluminescence and photoluminescence stability measurements (excitation at λ = 430 nm) were analyzed in a fluorometer (Edinburgh FLS1000) fitted with a 450 W ozone-free Xe arc lamp source in conjunction with a monochromator and a red PMT-900 detector. An integrating sphere set was employed for absolute PLQY measurements. Time-resolved photoluminescence measurements were performed with a nanosecond pulsed diode laser (435 nm) as an excitation source along with a time-correlated single-photon counting (TCSPC) detector.

Decay curves were fitted in a non-single-exponential stretched distribution^68^
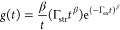
3where average decay time is equal to
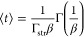
4Temperature-dependent photoluminescence measurements were acquired using a cryostat (Optistat-DM, Oxford Instruments) attached to a fluorometer. Temperature setting was automated by a remote controller (MercuryITC). Total transmittance and total reflectance measurements were obtained between the 350–700 nm range of the spectra in a Cary 5000 spectrophotometer (UV–vis–NIR) prepared with an internal DRA-2500 (PMT/PbS version).

## Data Availability

The data that support the findings of this study are openly available in Digital CSIC repository at https://doi.org/10.20350/digitalCSIC/15426.

## Supporting Information Available

Transmission electron microscopy cross-sectional images of the perovskite QD-embedded porous films, photoluminescence spectra of films embedding QDs with different sizes, effect of the perovskite synthesis parameters on the final PLQY achieved, and analysis of the temperature dependence of PL for films embedding QDs with different sizes (PDF)
