# Supplementary material for: Ultrapure Green High Photoluminescence Quantum Yield from FAPbBr3 Nanocrystals Embedded in Transparent Porous Films
Source: Chem Mater. 2023 Jul 7;35(14):5541–9. doi: 10.1021/acs.chemmater.3c00934 (PMC10389805; doi:10.1021/acs.chemmater.3c00934)
Supplement: Supplementary file 1 — cm3c00934_si_001.pdf [file cm3c00934_si_001.pdf]

Ultrapure Green High Photoluminescence Quantum Yield from $FAPbBr_3$ Nanocrystals Embedded in Transparent Porous Films.

Carlos Romero-Pérez[1], Natalia Fernández Delgado[2], Miriam Herrera-Collado[2], Mauricio E. Calvo[1,], Hernán Míguez[1,*]*

[1]Instituto de Ciencias de Materiales de Sevilla (Consejo Superior de Investigaciones Científicas-Universidad de Sevilla), C/Américo Vespucio, 49, Sevilla, 41092, Spain.

**E-mail:* mauricio.calvo@csic.es, h.miguez@csic.es

[2] Department of Material Science, Metallurgical Engineering and Inorganic Chemistry IMEYMAT, Facultad de Ciencias (Universidad de Cádiz), Campus Río San Pedro, s/n, Puerto Real, Cádiz, 11510, Spain.

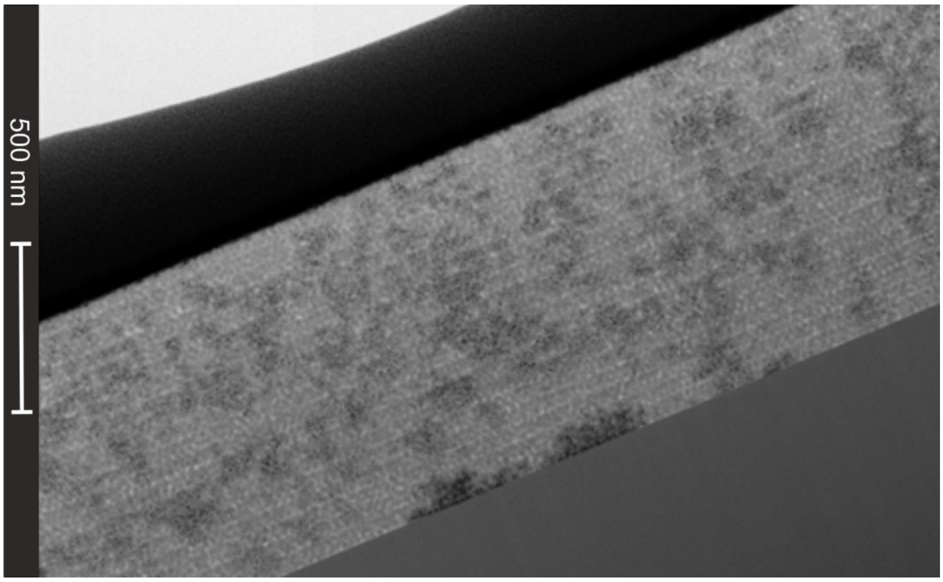

Figure S1. TEM micrograph from a lamella of a 1000 nm thick scaffold containing $FAPbBr_3$ QD within the pores. In this image, perovskite nanocrystals are distinguished as dark regions whereas 30nm size SiO_2 nanoparticles appear in pale gray and stratified as thin layers. It can also be observed that the careful control of the spin coating speed prevents the formation of an overlayer of bulky $FAPbBr_3$ on top of the porous film.

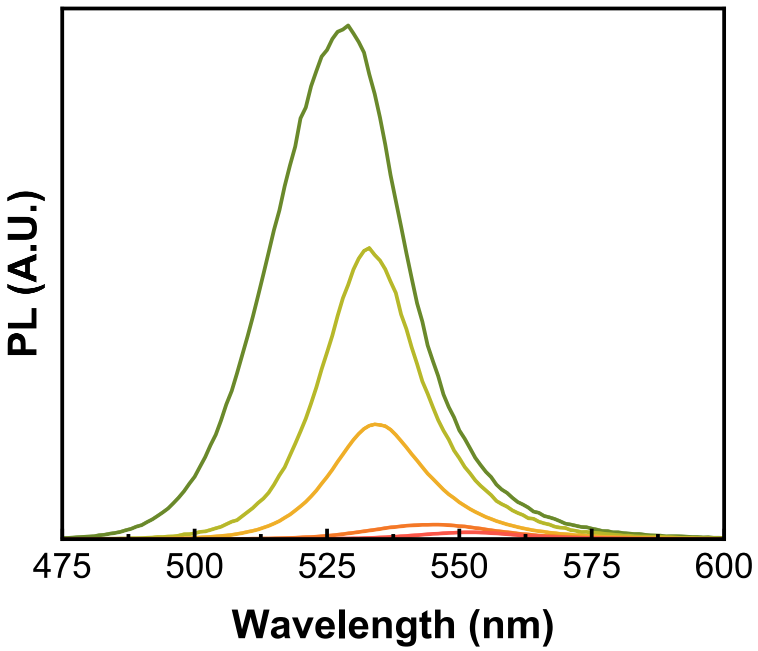

Figure S2. PL of $FAPbBr_3$ QD-SiO_2 films of 8.7 (olive), 10.2 (light green), 11.0 (yellow), 13.3 nm size (orange) and bulk film (pale red).

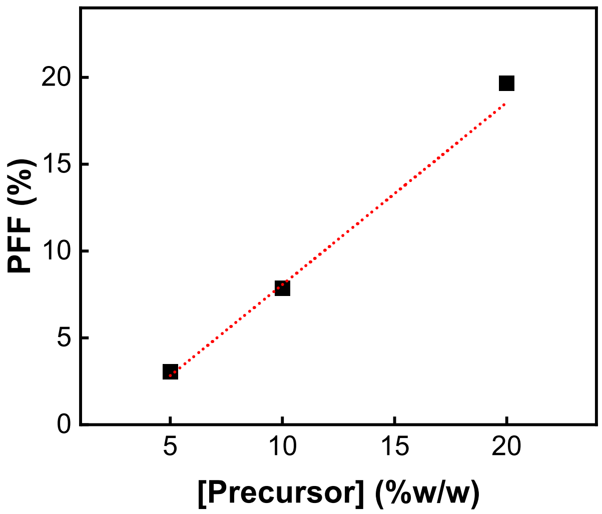

Figure S3. Pore filling fraction (PFF) of $FAPbBr_3$ sample prepared with 5, 10, 20% w/w solutions calculated from ICP measurements considering a ≅1000nm thick silica scaffold with a 50% volumetric porosity. (R=0.9923)

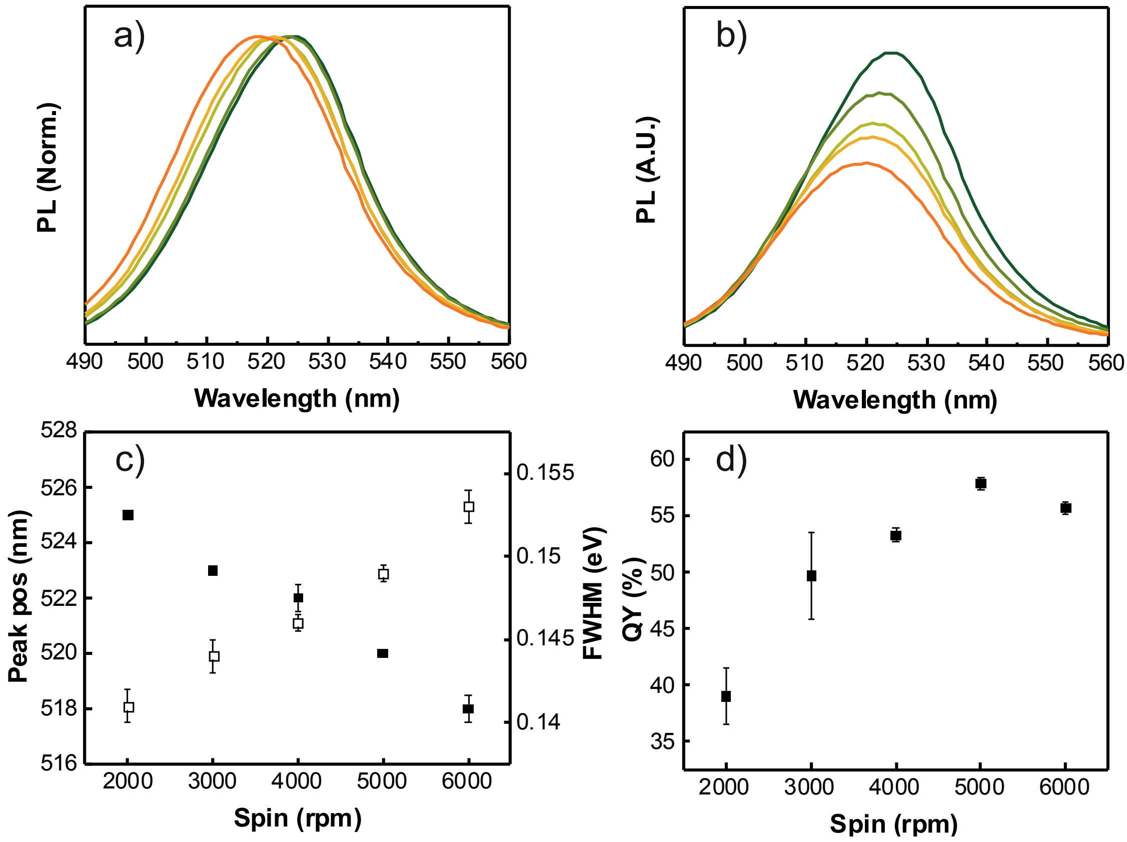

Figure S4. PL (a) and normalized PL (b) curves of $FAPbBr_3@SiO_2$ QD films for a fixed concentration in precursor solution (5% w/w) with a spin coating speed of 2000 (dark green), 3000 (olive), 4000 (light green), 5000 (yellow) and 6000 rpm (orange). (c) Peak position (full squares), FWHM (hollow squares) and (d) QY analysis of the previous samples.

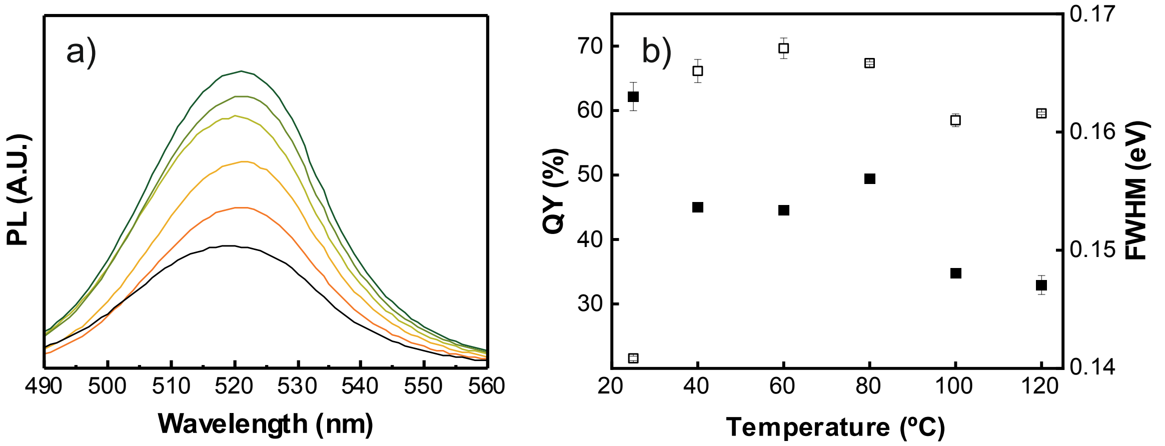

Figure S5. PL (a) curves of $FAPbBr_3@SiO_2$ QD films for a fixed concentration in precursor solution (5% w/w) with a synthesis temperature of 25 (black) 40 (dark green), 60 (olive), 80 (light green), 100 (yellow) and 120ºC (orange). (b) QY (hollow squares), and FWHM (full squares) analysis from samples the previous samples.

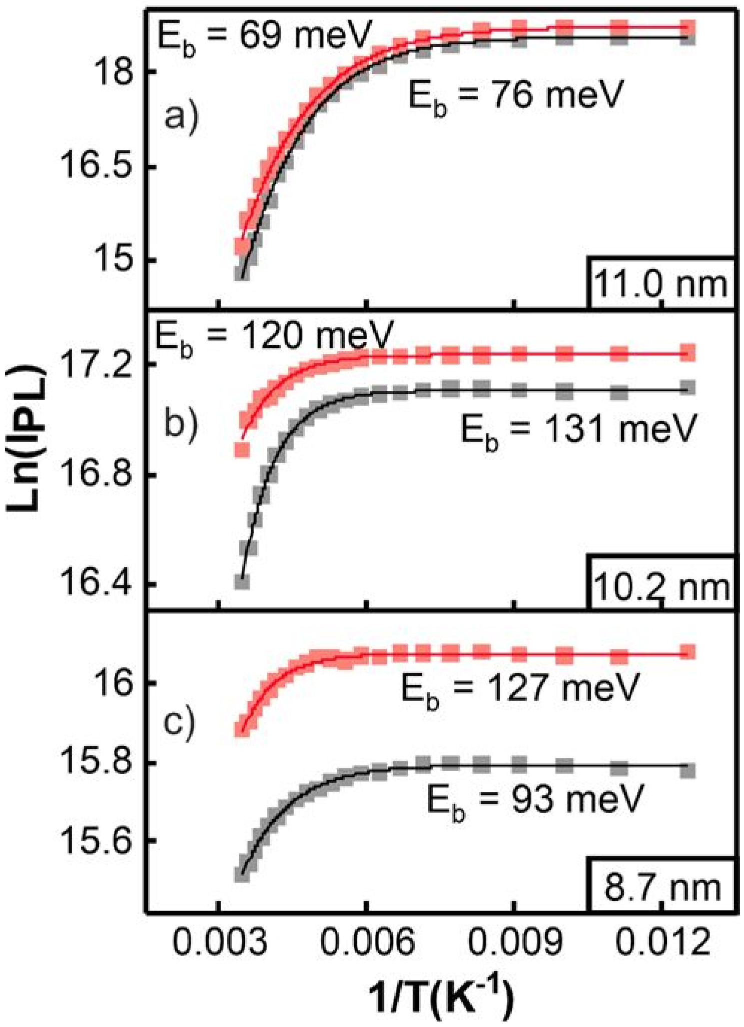

Figure S6. Temperature-dependent integrated photoluminescence intensity of $FAPbBr_3$ QD-SiO_2 films with 11.0 nm (a), 10.2 nm, (b) and 8.7 nm (c) QD size. Samples with and without PMMA are represented in pale red and gray full squares respectively. Arrhenius equation fitting for the integrated photoluminescence intensity is included following the same color code for each set of measurements.

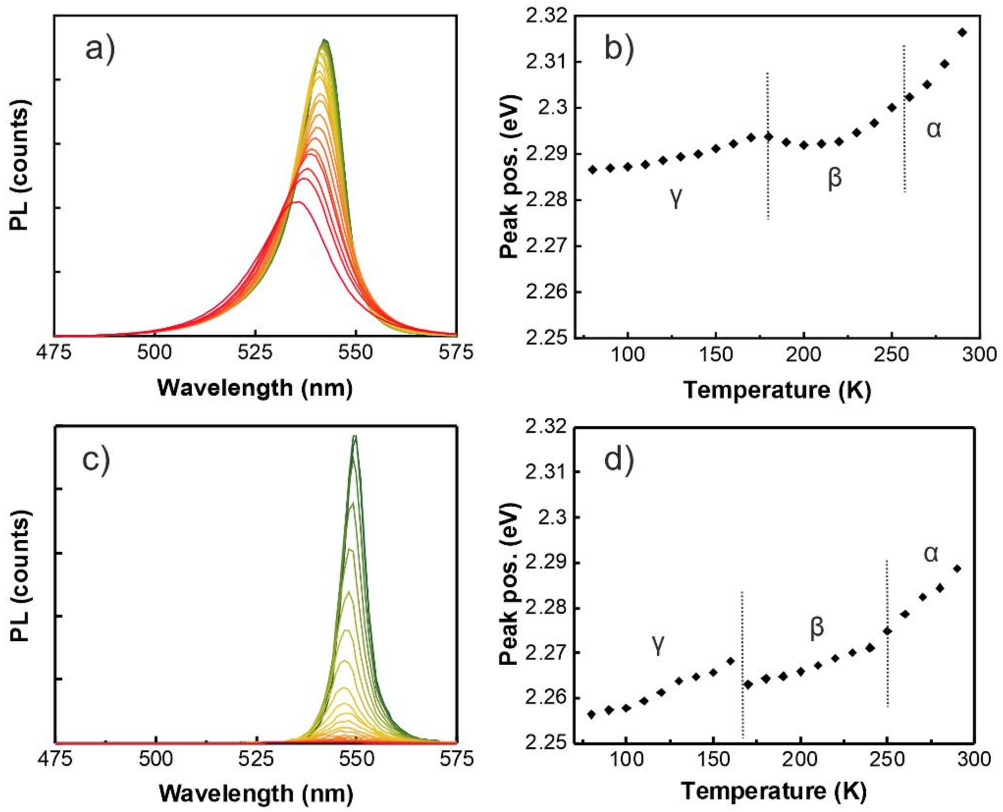

Fig S7. Temperature-dependent PL and PL maximum for 10.2 (a,b) and 11.0 nm (c,d) QD sizes from 80K (red curve) to 290K (green curve). Dashed lines in b) and d) are just a guide to the eye to separate different crystalline phases.

PMMA in CB (%w/w)	Peak pos. (nm)	FWHM (eV)	Lifetime (ns)	β	QY (%)
0	520	0.149	8.98	0.52	66
1	521	0.148	7.56	0.50	60
5	526	0.107	18.20	0.63	82
10	528	0.097	21.23	0.68	86

Table S1. PL peak and Time-resolved PL analysis of $FAPbBr_3$ QD@SiO_2 films w/ and w/o PMMA using a Stretched-exponential decay model.

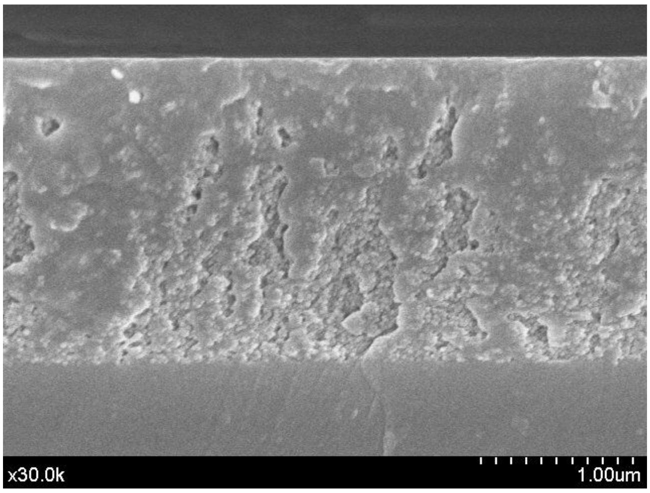

Figure S8. SEM micrograph from a 2 μm thick scaffold containing PMMA passivated $FAPbBr_3$ QD within the pores.

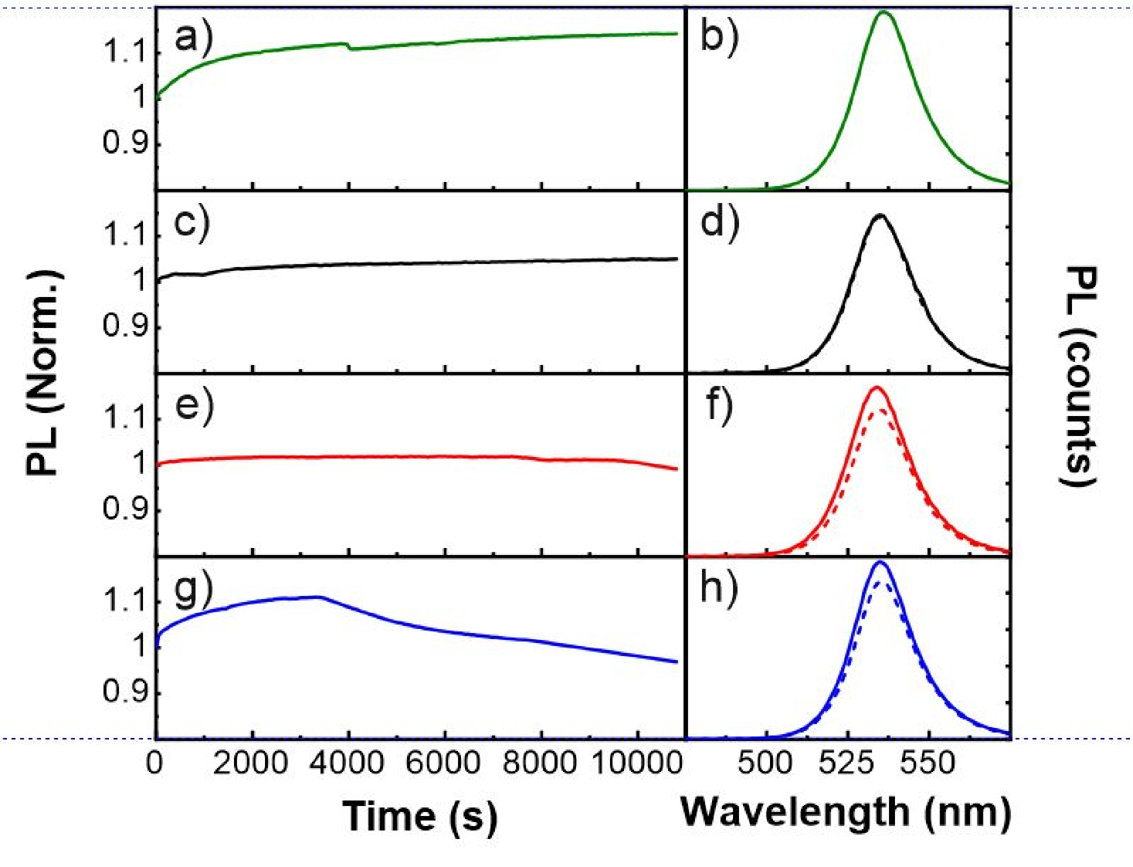

Figure S9. PL intensity normalized at t=0s recorded at the maximum wavelength emission and under constant illumination at λ=430nm at relative humidity RH= 40% (a), 55% (c), 70% (e), 80%(g). Initial (continuous line) and final (dashed line) spectra recorded at RH= 40% (b), 55% (d), 70% (f), 80%(h).
